# Plasma Glutathione Concentrations Are Associated with Leukocyte and Neutrophils’ Counts in Clozapine-Treated Patients

**DOI:** 10.3390/ph18091345

**Published:** 2025-09-08

**Authors:** Erick José Martínez-Rodríguez, Verónica Barón-Flores, Jesús Ramirez-Bermudez, Carlos Aviña-Cervantes, Dinora González-Esquivel, Araceli Diaz-Ruiz, Camilo Ríos

**Affiliations:** 1 Doctorado en Ciencias Biológicas y de la Salud, Universidad Autónoma Metropolitana, Ciudad de México 04960, Mexico; erick_martinezrodriguez@yahoo.com; 2 Laboratorio de Neurofarmacología Molecular, Departamento de Sistemas Biológicos, Universidad Autónoma Metropolitana Unidad Xochimilco, Ciudad de México 04960, Mexico; vbaron@correo.xoc.uam.mx; 3 Departamento de Neuropsiquiatría, Instituto Nacional de Neurología y Neurocirugía Manuel Velasco Suarez, Ciudad de México 14370, Mexico; jesusramirezb@yahoo.com.mx (J.R.-B.); karloz2@hotmail.com (C.A.-C.); 4 Departamento de Neuroquímica, Instituto Nacional de Neurología y Neurocirugía Manuel Velasco Suarez, Ciudad de México 14269, Mexico; aradiaruiz@hotmail.com; 5 Jefe de la División de Neurociencias, Instituto Nacional de Rehabilitación Luis Guillermo Ibarra Ibarra, Ciudad de México 14389, Mexico

**Keywords:** schizophrenia patients, clozapine, glutathione reduced, neutropenia, leukocyte

## Abstract

Clozapine’s potential hematological toxicity, particularly its effect on white blood cell counts, is well documented and routinely monitored in clinical practice. In this study, a significant association was identified between reduced glutathione levels and neutrophil counts in patients undergoing clozapine treatment. Among the variables analyzed, glutathione concentration showed the strongest correlation with neutrophil levels, suggesting a potential role for antioxidant status in mediating clozapine’s hematological effects. **Objective**: The study aimed to evaluate the relationship between plasma concentrations of clozapine, its active metabolite N-desmethylclozapine, reduced glutathione, and treatment duration, in relation to total leukocyte and neutrophil counts in patients attending an outpatient psychiatric clinic. **Methods**: Plasma levels of clozapine, N-desmethylclozapine, and reduced glutathione were quantified using validated analytical techniques. Complete blood counts were obtained, and a multiple regression analysis was conducted to identify factors most strongly associated with variations in neutrophil count. **Results**: Reduced glutathione levels were significantly associated with neutrophil counts (*p* = 0.009), representing the most robust association among the variables examined. Clozapine concentration and duration of treatment were also found to be relevant contributors to changes in hematological parameters. **Conclusions**: These findings suggest that individual antioxidant capacity, particularly involving glutathione metabolism, may influence susceptibility to clozapine-related neutropenia. This insight could inform future strategies for monitoring and managing clozapine-treated patients, potentially aiding in the identification of those at increased risk for hematological side effects.

## 1. Introduction

The prevalence of mental illness has shown an increase in recent years. In the study by Ritchie et al., it was estimated that in 2017, there were 792 million people in the world with a mental health illness [1]. Among them, schizophrenia is considered one of the most disabling medical disorders, and according to the World Health Organization, it is one of the ten diseases that contribute the most to the global burden [1,2,3]. It is important to highlight that a timely diagnosis and adequate treatment are essential to reduce symptoms and to provide patients with a good quality of life [4]. Currently, antipsychotic medications are considered the first line of treatment for schizophrenia. However, in some cases, adverse effects are observed, or patients develop resistance to treatment [5,6]. In this regard, treatment-resistant schizophrenia (TRS) is common, even in patients with first-episode schizophrenia in whom it has been reported that between 20 and 25% do not respond to adequate treatments with a first and/or second-line antipsychotic drug, despite having adequate treatment regimens, while between 10 and 20% of patients will develop TRS after initially responding to antipsychotic treatment [7]. It is important to note that this TRS population has a prevalence of 50 to 60% according to the report by Beck et al. [8]. Under this scenario, it is important to provide treatment options for patients with TRS. Clozapine is the only antipsychotic approved for this condition and for schizophrenia with persistent suicidal behavior [9]. Although there is a large amount of evidence supporting its efficacy and favorable risk-benefit ratio in patients who have failed two or more antipsychotic drugs, clozapine remains sub-utilized [10]. Clozapine was approved for the treatment of schizophrenia in 1990 by the Food and Drug Administration (FDA), given its therapeutic success [11]. Clozapine is well-absorbed, with peak plasma concentrations occurring approximately 2.5 h post-administration. It is extensively metabolized in the liver by cytochrome P450 enzymes, resulting in several metabolites, including N-desmethylclozapine, which also possess pharmacological activity. Clozapine exhibits a high affinity for various neurotransmitter receptors, including dopamine D4, serotonin 5-HT2A, and muscarinic receptors. Its unique receptor profile contributes to its efficacy in treatment-resistant schizophrenia and a lower incidence of extrapyramidal side effects compared to other antipsychotics.

Approximately 1% of patients may develop clozapine-induced agranulocytosis, typically within the first month of therapy [12]. Other rare but serious adverse effects include myocarditis and cardiomyopathy, which can occur after months or years, both of which are frequently less than 1% and probably not dose-dependent [13,14]. Other adverse effects reported are an increase in body weight [15], gastrointestinal hypomotility leading to constipation [16], and neutropenia [17]. In this regard, several studies have shown that clozapine-associated neutropenia occurs during the first 18 weeks; some cases occur after 6 months of treatment [18,19]. In some studies, changes in reduced glutathione (GSH) concentration have been reported between the intermediate phases of treatment [20]. Glutathione levels, typically measured in blood plasma, red blood cells, or brain tissue using techniques such as high-performance liquid chromatography or magnetic resonance spectroscopy, serve as a key biomarker of oxidative stress. Reduced GSH levels have been associated with a variety of neuropsychiatric disorders, including schizophrenia, indicating impaired antioxidant defense and redox imbalance.

In addition, a meta-analysis showed that the cumulative incidence of neutropenia is associated with the duration of clozapine treatment [21]. Severe neutropenia was found to be a rare event, but it can occur during the first 12 months of treatment [22]. Although the pathophysiological mechanism of neutropenia under clozapine treatment is uncertain, some studies have shown that N-desmethylclozapine can be toxic to granulocyte progenitor cells at concentrations three to six times higher than those normally achieved during treatment [23]. Uetrecht et al. [24] demonstrated that clozapine can be metabolized through the action of myeloperoxidase, generating nitrenium ion, a highly reactive metabolite. This mechanism may explain why clozapine and its metabolites exclusively affect the population of granulocytes and their precursors [12]. Another possible mechanism of damage is the irreversible binding of the nitrenium ion to the cell membrane structures of granulocytes, leading to the formation of neoantigens that, in turn, can provoke an immune response [12,25]. There is also evidence showing that patients with schizophrenia present functional alterations at the mitochondrial level, glutathione deficiency, and an increased formation of reactive oxygen species [26]. Emerging evidence suggests that GSH may influence clozapine’s pharmacokinetics and pharmacodynamics. GSH plays a role in the metabolism of various drugs, including antipsychotics, by modulating the activity of cytochrome P450 enzymes. Therefore, alterations in GSH levels could potentially affect the metabolism of clozapine, influencing its plasma concentrations and therapeutic efficacy. Furthermore, oxidative stress, which is heightened in schizophrenia, may impact the pharmacodynamics of clozapine by altering receptor function and neurotransmitter systems. The interplay between GSH, oxidative stress, and clozapine’s action warrants further exploration to better understand its therapeutic and adverse effects.

**Scheme 1:**
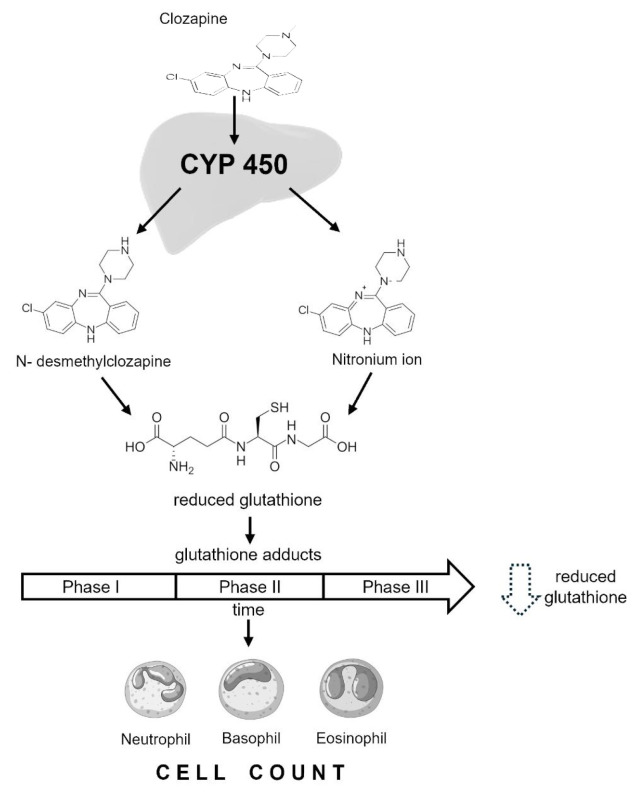
The underlying biological mechanism of clozapine-induced neutropenia and the protective role of glutathione are visualized. It begins with clozapine exposure. Next, the metabolism of clozapine by cytochrome P450 enzymes is shown, leading to the formation of reactive nitrenium ions and N-desmethylclozapine, represented using molecular icons. These reactive species trigger oxidative stress. In response, reduced glutathione acts as a protective antioxidant, neutralizing the reactive species and mitigating cellular damage, represented by a shield icon intercepting the stress molecules. However, in cases of reduced glutathione deficiency, oxidative stress persists, which may result in greater cellular damage. This may lead to the destruction of hematopoietic precursor cells, depicted as damaged cells, ultimately causing a decrease in neutrophil count.

Given the well-established therapeutic efficacy of clozapine, it remains the gold standard treatment for patients diagnosed with treatment-resistant schizophrenia, as well as for individuals presenting with persistent suicidal behavior associated with this mental disorder. Clozapine is often prescribed when other antipsychotic medications have failed to provide sufficient clinical improvement, making it an essential component in the management of a considerable proportion of the schizophrenia population. However, despite its clinical advantages, clozapine use is not without risks. One of the most concerning adverse effects is its potential to induce hematological abnormalities, particularly reductions in leukocyte and neutrophil counts, which can lead to life-threatening conditions such as agranulocytosis. These side effects necessitate regular blood monitoring and limit its broader use. In light of these challenges, the present study aimed to explore the possible association between reduced glutathione levels, an important endogenous antioxidant, and white blood cell parameters. Specifically, this research seeks to investigate whether it plays a contributory role in the development or modulation of clozapine-induced hematological alterations, offering insights into underlying protective or risk mechanisms. Scheme 1 visually illustrates the biological mechanism underlying clozapine-induced neutropenia and the protective role of glutathione.

## 2. Results

Fifty-nine patients with a diagnosis of schizophrenia, treated with clozapine, were studied. The demographic and descriptive characteristics of the studied population are shown in Table 1. Forty-one (69.5%) men and eighteen (30.5%) women were included in the study. The age ranged from 18 to 69 years. Only the clozapine treatment duration was significantly different between genders, *p* ≤ 0.04.

**Figure 1:**
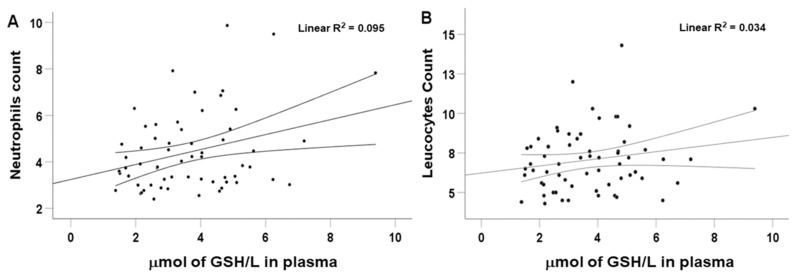
Scatter plot showing the main relationships between neutrophil counts (panel (**A**)) and leucocyte counts (panel (**B**)) vs. plasma GSH concentration. Pearson’s correlation and linear regression analysis were applied. An association between both variables was determined with an R^2^ of 0.095 and 0.034, respectively.

Figure 1 shows the scatter plot of the neutrophil and leukocyte counts as a function of the GSH plasma concentration. A Pearson correlation coefficient of 0.307 and 0.184 was determined with a statistical significance of *p* < 0.05, and a positive association between both variables was observed with an R^2^ of 0.095 and 0.034 for neutrophils and leukocytes, respectively.

**Figure 2:**
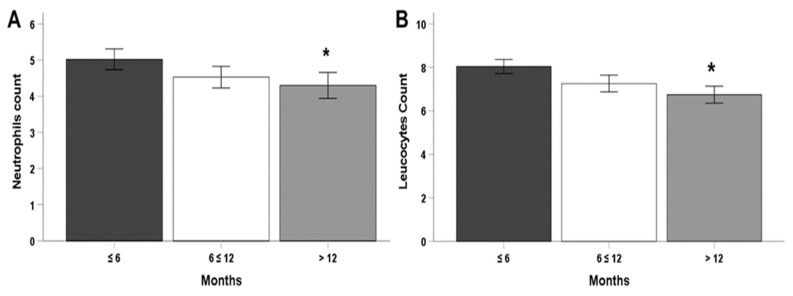
Neutrophil and leukocyte counts, panels (**A**) and (**B**), respectively, by clozapine treatment phase. * Different from Phase 1; *p* < 0.05, ANOVA followed by post hoc Tukey’s test.

Figure 2 shows, in panel (A), the neutrophil and, in panel (B), the leukocyte counts as a function of the duration of clozapine treatment phases. A clear trend to decrease both counts as clozapine treatment duration increases is observed. Neutrophils’ values (phase 1, 5021 ± 289; phase 2, 4530 ± 299; phase 3, 4300 ± 359), and leukocytes’ counts (phase 1, 8044 ± 321; phase 2, 7259 ± 388; phase 3, 6748 ± 390). Significance was attained only when comparing phase 1 and phase 3 means for both counting methods.

Table 2 shows the results of the multiple linear regression model. It was observed that the model with the best fit has an R^2^ of 26.5% as a predictor of the neutrophil counts, an observed statistical power of 0.967, and an R^2^ of 19.5% as a determinant of leukocytes. Table 2 shows the determinant variables with their respective coefficients that were statistically significant in that model (*p* < 0.05).

**Figure 3:**
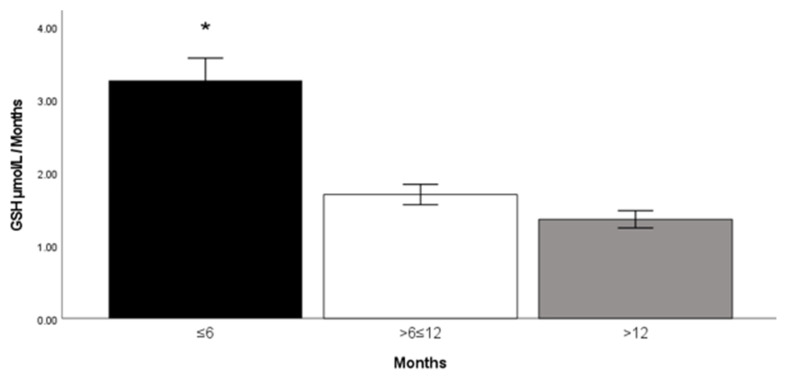
Comparison of levels of reduced glutathione (GSH) by treatment phase in patients under clozapine. * Different from phases 2 and 3; *p* < 0.001. ANOVA followed by post hoc Tukey’s test.

Figure 3 shows the changes in plasma GSH along the phases (time). It is noteworthy that a remarkable decrease in mean plasma GSH concentrations (−48%) occurs after 6–12 months of clozapine treatment, suggesting that early-phase use of clozapine may be associated with a larger depletion of GSH, as after 12 months of treatment, GSH remains practically unchanged. It was also observed that GSH levels tended to decrease with increasing the time of clozapine use. An ANOVA analysis with Tukey’s post hoc test was performed to determine inter-phase differences of glutathione, showing statistically significant differences. The GSH/treatment phase ratio values decrease as clozapine use time increases, respectively (phase 1, 3.27 ± 0.97; phase 2, 1.7 ± 0.71; phase 3, 1.36 ± 0.61). Phase 1 showed to be statistically significant compared to phases 2 and 3 (*p* < 0.001).

**Figure 4:**
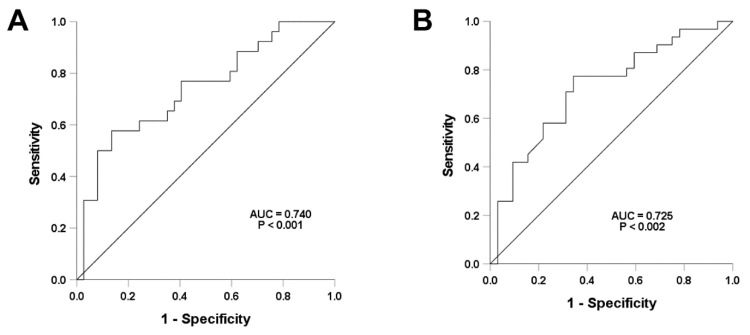
Panel (**A**) neutrophils ROC curve of GSH/treatment phase in patients receiving clozapine treatment, *p* < 0.001. Panel (**B**) leukocytes ROC curve of GSH/treatment phase in patients receiving clozapine treatment, *p* < 0.002.

Using a receiver operating characteristic (ROC) curve analysis, we propose a potential diagnostic value for evaluating the severity of neutropenia associated with clozapine treatment. Specifically, the area under the curve (AUC) was calculated for both neutrophil and leukocyte counts in relation to the ratio of reduced glutathione concentration to the number of months under treatment (GSH/treatment phase). This ratio demonstrated a promising predictive capacity, with an AUC of 0.74 (*p* < 0.001) for neutrophils and 0.72 (*p* < 0.002) for leukocytes, indicating a statistically significant association. These results suggest that the GSH/treatment phase ratio may serve as a useful marker for identifying patients at increased risk of developing clozapine-induced neutropenia. Further research with larger sample sizes is necessary to validate its clinical applicability. For a graphical representation of the ROC curves, refer to Figure 4.

## 3. Discussion

The principal finding of this study was the identification of a statistically significant association between plasma concentrations of reduced glutathione and the counts of both leukocytes and neutrophils in patients undergoing treatment with clozapine. This relationship suggests a potential role of oxidative stress and antioxidant defense mechanisms in the hematological changes observed in these individuals. To the best of our knowledge, this is the first report to document such an association in the context of clozapine therapy. In addition to this main finding, a notable difference was observed in the duration of clozapine treatment between male and female participants, as detailed in Table 1. Furthermore, while both neutrophil and leukocyte counts showed variability across different treatment durations, statistically significant differences were observed only at the longest time interval analyzed—specifically in patients who had been treated with clozapine for more than 12 months. These findings highlight the importance of monitoring treatment duration and individual patient characteristics when evaluating blood parameters. In the work of Matsui et al. [27], a decrease in the number of neutrophils and leukocytes was found in patients who use clozapine. In this study, no patient showed clinically significant neutropenia. However, Alvir et al. [18] reported that severe neutropenia, defined as a total neutrophil count of less than 500 cells/μL, develops in 0.8% of patients taking clozapine at one and a half years of treatment. Lambertenghi-Deliliers [19] found, in Italian patients, an incidence of 0.7% of neutropenia associated with the consumption of clozapine after one year of treatment. While Tang et al. [28] reported an incidence of 0.21% in China, where, unlike Western countries, clozapine is widely prescribed as the first line of therapy. All those studies agree with our present results.

Previous research has demonstrated that clozapine-associated neutropenia typically develops during the early stages of treatment, particularly within the first 18 weeks. However, there are documented cases in which neutropenia has appeared even after six months of continuous clozapine use. In those studies, significant differences in glutathione concentrations were observed between different treatment phases, suggesting that antioxidant status may fluctuate over time and could be related to hematological outcomes [18,29]. Furthermore, some studies are consistent with the findings of the present work. For instance, Ballesteros et al. [30] reported that patients treated with clozapine exhibited significantly lower levels of reduced glutathione compared to those not receiving the drug. This supports the hypothesis that glutathione plays a role in clozapine-induced neutropenia. Nevertheless, despite these associations, none of the available studies, including those mentioned, have established a definitive cutoff value for glutathione concentration that could reliably predict the onset or risk of neutropenia in clozapine-treated patients.

Raffa et al. [20] conducted a comparative study involving patients diagnosed with schizophrenia, analyzing two distinct groups: those undergoing pharmacological treatment and those who were not receiving any antipsychotic medication at the time of the study. Their findings revealed a significant reduction in glutathione levels, as well as a marked decrease in the activity of key antioxidant enzymes, in the group receiving pharmacological treatment. These results suggest that antipsychotic medications, including clozapine, may influence the redox balance and oxidative stress pathways in patients, potentially contributing to cellular damage and adverse effects. Moreover, the study adds valuable evidence supporting the role of reduced glutathione as a potential biological marker, not only for monitoring oxidative status but also for assessing the severity of adverse drug reactions. As such, glutathione levels may serve as an early indicator of vulnerability to treatment-related complications, highlighting the importance of incorporating oxidative stress biomarkers into the clinical evaluation and follow-up of patients with schizophrenia.

There is substantial evidence indicating that patients diagnosed with schizophrenia exhibit significantly reduced activities of key antioxidant enzymes, including glutathione peroxidase, superoxide dismutase, and catalase. This reduction in enzymatic activity compromises the body’s natural defense mechanisms against oxidative stress, rendering these individuals more susceptible to the damaging effects of reactive oxygen species, particularly those generated as metabolites during clozapine treatment. The resulting imbalance between oxidative agents and antioxidant defenses may contribute not only to the pathophysiology of schizophrenia, but also to the occurrence of adverse effects linked to antipsychotic therapy. In this context, Zhang et al. [31] conducted a comparative study in which they demonstrated that the enzymatic activities of superoxide dismutase and glutathione peroxidase were significantly lower in patients with schizophrenia compared to healthy control subjects. Based on their findings, the authors concluded that oxidative stress plays a relevant role in the underlying mechanisms of schizophrenia. Moreover, they suggested that treatment with clozapine may exacerbate this oxidative imbalance by further inhibiting antioxidant enzyme activity, potentially contributing to treatment-related toxicities.

Some studies, such as the one conducted by Hendouei et al. [32], have reported that clozapine may exert antioxidant effects under certain conditions. In their comparative study, the authors evaluated the effects of clozapine versus risperidone in patients with schizophrenia and found that clozapine treatment led to an increase in superoxide dismutase activity and elevated levels of reduced glutathione. Additionally, clozapine was associated with a reduction in lipid peroxidation, suggesting a potential protective role against oxidative damage. However, despite these findings, it is well-established that clozapine can also induce agranulocytosis through various mechanisms, including the generation of reactive oxygen species such as the nitrenium ion [33], which may contribute to cellular toxicity and hematological adverse effects.

In this study, no statistically significant differences were observed in the plasma concentrations of N-desmethylclozapine between male and female participants. Although it is well documented that clozapine metabolism may contribute to the development of agranulocytosis, the exact underlying mechanisms remain poorly understood. One proposed hypothesis suggests that the metabolic biotransformation of clozapine can lead to the formation of reactive oxygen species, including highly reactive intermediates such as nitrenium ions, which may exert cytotoxic effects on bone marrow cells. This oxidative stress may ultimately contribute to the reduction in leukocyte and neutrophil counts observed in some patients undergoing long-term treatment with clozapine.

In our multiple linear regression analysis, several variables were evaluated to identify their predictive value in relation to white blood cell parameters.

The final model included duration of treatment and glutathione levels, which were statistically significant predictors of neutrophil and leukocyte counts. Plasma clozapine concentration was also included due to its borderline significance and clinical relevance, despite not reaching conventional statistical significance.

Notably, reduced glutathione concentration emerged as the strongest and most consistent variable associated with decreased neutrophil and leukocyte levels. These findings underscore the potential role of oxidative stress and antioxidant depletion in the hematological adverse effects observed in patients receiving clozapine, suggesting that glutathione monitoring could be valuable in clinical practice. Our hypothesis is based on the assumption that patients undergoing treatment with clozapine, who exhibit lower levels of reduced glutathione, may face an increased risk of experiencing a significant reduction in neutrophil and leukocyte counts. Reduced glutathione plays a crucial role as a potent antioxidant that helps protect cells from oxidative damage, particularly in individuals receiving long-term pharmacological treatment with clozapine. Given that clozapine has been shown to potentially increase oxidative stress by generating reactive oxygen species, it is plausible that patients with a pre-existing depletion in antioxidant defense mechanisms, such as low glutathione levels, could be more susceptible to hematological alterations. These alterations, which include neutropenia and leukopenia, may result in an increased vulnerability to infections and other complications. Thus, we hypothesize that monitoring reduced glutathione levels in patients treated with clozapine could serve as an early indicator of hematological risk, enabling more personalized and proactive clinical management strategies.

The ROC curve analysis conducted in this study suggests that reduced glutathione levels could potentially serve as a diagnostic tool for assessing the severity of neutropenia in patients undergoing clozapine treatment. A decrease in reduced glutathione concentration was found to be strongly associated with a reduction in neutrophil and leukocyte counts. However, while these findings are promising, further validation through larger sample sizes and additional research is needed before definitive conclusions can be drawn. Future studies should aim to confirm or refute the proposed relationship between glutathione levels and the severity of neutropenia, as well as explore the potential utility of this marker in clinical practice. At present, to mitigate the risk of neutropenia in patients taking clozapine, regular hematological monitoring has become a mandatory practice in many countries. For example, in the United Kingdom, complete blood counts (CBCs) are ordered weekly during the first 18 weeks of treatment, followed by biweekly CBCs until week 52, and then monthly for the duration of the patient’s treatment (https://bnf.nice.org.uk/drug/clozapine, accessed on 6 January 2020). This structured monitoring approach has proven effective in identifying neutropenia early, which is crucial for preventing serious complications, including agranulocytosis.

In light of our findings, the ratio calculated by dividing reduced glutathione concentration by the treatment phase (GSH/Treatment Phase) may hold diagnostic value for the ongoing monitoring and evaluation of patients on clozapine therapy. However, it is important to emphasize that this hypothesis remains speculative until further studies are conducted to confirm its clinical relevance and practicality. The potential role of reduced glutathione as a predictive biomarker for clozapine-induced neutropenia could offer a valuable addition to current monitoring protocols, improving the safety and management of patients receiving this treatment.

Given the observed association between reduced glutathione levels and neutrophil counts in clozapine-treated patients, strategies aimed at modulating GSH concentrations may offer therapeutic benefits. Several interventions have been investigated to restore or enhance GSH levels in neuropsychiatric disorders. Among them, N-acetylcysteine, a precursor to cysteine and a direct GSH donor, has shown promise in schizophrenia, not only for its antioxidant effects, but also for its potential to improve negative symptoms and cognitive function.

### Study Limitations

This study has several limitations that should be considered. Its cross-sectional design prevents the establishment of causal relationships between glutathione concentrations, clozapine levels, and hematological parameters. Although significant associations were identified, the directionality of these effects cannot be determined. The sample size, while adequate for exploratory analyses, was limited once stratified by treatment duration, potentially reducing statistical power.

All participants were recruited from a single specialized center in Mexico City, which may restrict the generalizability of findings to other populations with different genetic, environmental, or nutritional characteristics. Additionally, potential confounding factors—such as nutritional status, comorbid physical conditions, and the use of concomitant medications—were not systematically controlled, and these may influence both glutathione metabolism and blood cell counts.

Plasma glutathione and clozapine levels were measured at a single time point, without accounting for possible circadian or pharmacokinetic variability. Moreover, other oxidative stress markers were not included, limiting the interpretation of glutathione levels within the broader pathophysiological context. These limitations highlight the need for future longitudinal and multicenter studies, with more comprehensive biochemical profiling, to validate and expand upon these findings.

## 4. Materials and Methods

### 4.1. Study Design and Population

This cross-sectional clinical study included 59 patients diagnosed with schizophrenia, all of whom were receiving clozapine as their primary antipsychotic treatment. Participants were recruited from the Neuropsychiatry Clinic of the National Institute of Neurology and Neurosurgery in Mexico City. The study was conducted in accordance with the Declaration of Helsinki (October 1996 version) and Good Clinical Practice guidelines. Approval was obtained from the Institutional Ethics Committee (Registry No. 92/07), and all participants provided written informed consent following a full explanation of the study’s purpose and procedures. The manuscript was prepared following the STROBE guidelines [34]. Inclusion criteria were: (1) age ≥ 18 years, (2) a diagnosis of schizophrenia according to DSM-5 criteria, and (3) being under stable clozapine treatment. Exclusion criteria included: a non-schizophrenia diagnosis, concomitant neurological disorders, alcohol or substance dependence within the past six months, pregnancy or lactation, and refusal to participate. Patients were stratified by treatment duration into three phases: Phase 1: ≤6 months (*n* = 18), Phase 2: >6 months and ≤12 months (*n* = 20), and Phase 3: >12 months (*n* = 21). The main variables analyzed were clozapine dose, treatment duration (years), plasma clozapine and N-desmethylclozapine levels, plasma reduced glutathione (GSH), and leukocyte and neutrophil counts.

### 4.2. Determination of Plasma Clozapine and N-Desmethylclozapine

For the determination of clozapine and N-desmethylclozapine, an analytical method by high-resolution liquid chromatography with ultraviolet detection was optimized and validated, based on the works reported by Frahnert et al., Llerena et al., Titier et al. [35,36,37]. The wavelength used for the detection of clozapine and desmethylclozapine was 220 nm. A Brava-o-CN column (250 mm length, 4.6 mm internal diameter, and 5 µm particle size, Agilent Technologies, Santa Clara, CA, USA) was used, and a precolumn of the same material and the same particle size as the Brava CN column was used.

The mobile phase consisted of acetonitrile/water/1 M ammonium acetate (50:49:1 *v*/*v*), with a pH of 5.5 at a flow rate of 1 mL/min. The elution time was 20 min, and the retention times were: 14.9 min for N-desmethylclozapine, 17.9 min for clozapine, and 19.6 min for loxapine, which was used as an internal standard [38]. The HPLC-UV method was linear, exact, had intra- and inter-day precision, and was stable to measure concentrations of CLZ between 30 and 1000 ng/mL, while 12.5–560 ng/mL of the metabolite. It was a selective method and also allowed the adequate resolution of internal standard, from CLZ and N-desmethylclozapine.

### 4.3. Determination of Reduced Glutathione (GSH)

The concentration of GSH was determined using a fluorometric method reported by Hissin and Hilf [39]. The method is based on the reaction of GSH with o-phthalaldehyde (OPA) to form a highly stable and fluorescent indole derivative. The blood sample was centrifuged at 3000 rpm for 10 min. In total, 200 µL of serum was mixed with 200 µL of 25 percent (*w*/*v*) metaphosphoric acid. The tubes were then placed on ice and centrifuged at 12,500 rpm for 20 min at 4 °C. In total, 20 µL aliquots of the supernatant were placed in a tube and added with 3980 µL of 0.05–0.1 M EDTA-phosphate buffer. Additionally, 100-µL of the previous mixture was taken and placed with 1800 µL of 0.05–0.1 M EDTA-phosphate buffer. Furthermore, 100 µL of an o-phthaldehyde solution, at a concentration of 7.4 μM, was added. The mixture was incubated for 15 min for later reading. Fluorescent signals were measured in a luminescence spectrophotometer (Perkin Elmer LS50B, Shelton, CT, USA) at 370 nm excitation and 420 nm emission. Calibration curves were constructed in a range of 4.5–72 μmol. A 600 μmol GSH stock solution was used. The concentrations were obtained by interpolation on the standard curve. Results were expressed as µmol GSH/L.

### 4.4. Hematological Analysis

The counts of neutrophils and leukocytes were carried out at the clinical laboratory of the National Institute of Neurology and Neurosurgery. Blood samples from all participants were collected and processed following standard procedures to ensure accuracy and consistency. These samples were subsequently analyzed using the XN-1000 Sysmex hematology analyzer (Sysmex, Irvine, CA, USA), a widely used and highly reliable instrument for hematological assessments. The analysis was performed according to the manufacturer’s protocol, which includes precise steps for sample preparation, calibration, and quality control to guarantee the validity of the results. This advanced analyzer enabled the accurate measurement of various blood components, including neutrophils and leukocytes, which are crucial for evaluating the hematological effects of clozapine treatment. The protocol ensured that the data obtained from the analysis were both reproducible and reliable, contributing to the overall robustness of the study’s findings.

### 4.5. Statistical Analyses

The variables studied were: clozapine concentrations, N-desmethylclozapine concentrations, concentration of reduced glutathione, years under clozapine treatment, clozapine daily dose, and neutrophil and leukocyte counting.

Normality was tested for all continuous variables, using the Kolmogorov–Smirnov test and homogeneity of variances with the Levene test. Student’s *t*-test was performed to compare the means of continuous variables. For the comparison between treatment phases, a one-way analysis of variance (ANOVA) was performed, followed by a post hoc Tukey’s test. A multiple linear regression analysis model was applied to the data to identify predictor variables with the best fit for the model. After identifying those variables, a ROC curve was designed to evaluate the diagnostic value of the quotient: GSH concentration/treatment phase, using the mean value of the leukocytes and neutrophils’ counting in a categorical variable, and a cut-off point (neutrophils 4.143/mL and leukocytes 6.708/mL). The differences were considered statistically significant when *p* < 0.05. Data were analyzed using SPSS version 25.0.

## 5. Conclusions

In conclusion, our findings indicate that reduced glutathione concentration, along with treatment duration and blood clozapine concentrations, are key variables for predicting neutrophil and leukocyte counts in patients receiving clozapine. The observed association between decreased reduced glutathione and the reduction in these counts underscores the importance of monitoring this biomarker in clinical practice. Additionally, this study proposes a potential index for assessing the severity of neutropenia associated with clozapine use. However, further research with larger sample sizes is necessary to validate these results.

## Figures and Tables

**Table 1:** Descriptive statistics of 59 patients treated with CLZ.

Variable	Women	Men	*p*
Weight (kg)	65.47 ± 7.59	83.91 ± 15.03	0.00
Age (years)	37.9 ± 10	35.17 ± 9.68	NS
Clozapine dosage (mg/day)	255.88 ± 32.33	276.54 ± 28.15	NS
Neutrophils (×10^3^/mL)	4.143 ± 1.566	4.735 ± 1.730	NS
Leukocytes (×10^3^/mL)	6.708 ± 2.144	7.470 ± 1.918	NS
Clozapine concentration (ng/mL)	537.32 ± 82.31	447.144 ± 43.07	NS
N-desmethylclozapine concentration (ng/mL)	152.507 ± 32.1	173.96 ± 28.96	NS
GSH concentration (μmol/L)	4.13 ± 1.37	3.40 ± 1.66	NS
Years of clozapine treatment	6.47 ± 1.37	3.65 ± 0.61	0.04

GSH: reduced glutathione, data are presented as mean ± S.E. standard error. Continuous variables were used via Student’s *t*-test. NS: Not significant.

**Table 2:** Multiple linear regression models of neutrophils and leukocyte counts.

	(β)	*t*	*p*
Neutrophils			
Clozapine plasma levels	0.224	1.897	0.063
GSH concentration	0.327	2.691	0.009
Phase	−0.291	−2.443	0.018
Leucocytes			
GSH concentration	0.265	2.205	0.031
Phase	−0.384	−3.199	0.002

Reduced glutathione (GSH), standardized coefficient (β).

## Data Availability

The original contributions presented in this study are included in the article. Further inquiries can be directed to the corresponding authors.
